# Consent to discuss participation in research: a pilot study

**DOI:** 10.1136/ebmental-2019-300116

**Published:** 2019-09-26

**Authors:** Sophie Walker, Jennifer Potts, Lola Martos, Alvaro Barrera, Mark Hancock, Stuart Bell, John Geddes, Andrea Cipriani, Catherine Henshall

**Affiliations:** 1 Department of Psychiatry, University of Oxford, Oxford, UK; 2 Oxford Health NHS Foundation Trust, Warneford Hospital, Oxford, UK, Oxford, UK; 3 Faculty of Health and Life Sciences, Oxford Brookes University, Oxford, UK

**Keywords:** adult psychiatry

## Abstract

**Background:**

Equitable access to research studies needs to be increased for all patients. There is debate about which is the best approach to use to discuss participation in research in real-world clinical settings.

**Objective:**

We aimed to determine the feasibility of asking all clinical staff within one hospital Trust (an organisation that provides secondary health services within the English and Welsh National Health Service) to use a newly created form on the Trust’s electronic patient records system, as a means of asking patients to consent to discuss participation in research (the opt-in approach). We also aimed to collect feedback from patients and clinicians about their views of the opt-in approach.

**Methods:**

Four pilot sites were selected in the Trust: two memory clinics, an adult mental health team and an acute adult ward. Data were collected in three phases: (1) for 6 months, pilot site staff were asked to complete a consent to discuss participation in research form with patients; (2) staff feedback on the form was collected through an online survey; and (3) patient feedback was collected through focus groups.

**Findings:**

Of 1779 patients attending services during the pilot period, 197 (11%) had a form completed by staff and 143 (8%) opted-in to finding out about research. Staff cited limited time, low priority and poor user experience of the electronic patient records system as reasons for low uptake of the form. Patients generally approved of the approach but offered suggestions for improvement.

**Conclusions:**

There were mixed results for adopting an opt-in approach; uptake was very low, limiting its value as an effective strategy for improving access to research.

**Clinical implications:**

Alternative strategies to the opt-in approach, such as transparent opt out approaches, warrant consideration to maximise access to research within routine clinical care.

## Background

Patients who take part in research generally have improved clinical outcomes, while research active clinical services have lower mortality rates and also have superior overall clinical performance.^1^ However, recruitment to research within the UK National Health Service (NHS) is challenging and involves many barriers, especially in mental health.^2^ One key barrier is that researchers often are not permitted to contact patients directly about research due to concerns about confidentiality, data protection and patient vulnerability; this can have a detrimental impact on recruitment rates.^3^

Researchers are largely dependent on clinician referrals to recruit participants to studies, but these referrals are influenced by busy clinicians’ understanding of research study protocols and their attitudes and beliefs around the benefits of research.^4^ Clinicians who hold dual academic/clinical roles are more likely to refer patients to research studies,^5 6^ thus research participants often only represent patients from a small numbers of clinicians, rather than being representative of the general clinical population.

New strategies to improve access to research for all patients became a key priority for Oxford Health NHS Foundation Trust (OHFT), when it gained National Institute for Health Research Biomedical Research Centre status in 2017. A Trust is an organisation that provides secondary health services within the English and Welsh NHS. One potential strategy for increasing recruitment rates was to adopt an opt-in approach, in which the patient’s clinician seeks consent to discuss their participation in research (CDPR). To be successful, this process needs to ensure that all patients are properly informed about the benefits of research participation, as well as training all clinical staff to introduce research opportunities to patients.

Integrating an opt-in approach into clinical practice requires the recording of patient consent. At OHFT, an electronic patient records system (EPR) used by clinical staff within the trust was used for this purpose. A new form was added to EPR where CDPR could be recorded, thus simplifying the data collection process and improving access to the CDPR form for staff members.

The CDPR form (online supplementary appendix 1) was developed after consultation with key stakeholders, including representatives from Oxford Health Research & Development, clinicians and the Clinical Record Interactive System (CRIS) oversight committee (which included patient representatives). The CRIS system is a research platform across the UK that harnesses over 2 million deidentified patient records so that data can be used for research and audit purposes (https://crisnetwork.co/). The form collects information on whether patients’ consent to being contacted about research, including after their discharge. It also records whether patients’ have capacity to complete the CDPR form.

10.1136/ebmental-2019-300116.supp1 Supplementary data

To understand whether the form would be used by clinical staff, and whether this opt-in approach was valuable to patients and staff, we developed the current study, which also aimed to consider alternative strategies to engaging patients in research.

## Objective

Our objectives were: (1) to determine the feasibility of asking all clinical staff to use the CDPR form (measured as the proportion of patients who had a CDPR form completed on EPR) and (2) to collect feedback from staff and patients about their experiences and views on the value of the opt-in approach.

## Method

### Study design

This was a mixed methods study consisting of three phases: (1) quantitative data capture of the number of patients offered the opt-in approach by clinicians (via the CDPR form), (2) an online staff survey and (3) qualitative focus groups with patients. The study protocol is reported in online supplementary appendix 2.

10.1136/ebmental-2019-300116.supp2 Supplementary data

### Access, recruitment and setting

Four pilot sites were selected using convenience sampling. The sites were representative of a range of mental health clinical services and patients across the Trust: two memory clinics (MCs), an adult mental health team (AMHT) and an acute adult ward (AA). Each pilot site consisted of staff with diverse roles and varying levels of clinical expertise; all had access to EPR via an electronic device or computer.

Phase 1: during an introductory visit at each pilot site by researchers, staff were shown the location of the CDPR form on EPR, trained how and when to ask the opt-in question and were provided with CDPR leaflets with contact details of the CDPR study team. Staff were encouraged to develop their own implementation strategy regarding when they asked the opt-in question (eg, assessment visit, during treatment and discharge).

Phase 2: following completion of phase 1, all staff had participated were sent an online staff survey to elicit their views on the CDPR and opt-in approach.

Phase 3: UK-CRIS was used to search EPR to identify patients who had opted-in during phase 1. These patients were contacted by phone by a researcher (SW) about taking part in a focus group and, if interested, were sent a participant information leaflet by email or post. Due to a very low uptake of CDPR at one of the pilot sites, focus groups only took place at three of the sites. MC patients were invited to attend with a study partner/carer if they wished.

### Data collection

Data collection was undertaken sequentially between the three study phases. Phase 1 data were collected between July and December 2018, phase 2 data was collected between May and June 2019 and phase 3 data was collected in June 2019.

Phase 1: clinical staff discussed the opt-in approach with OHFT patients attending clinical services at each of the pilot sites, over 6 months. This excluded patients who lacked capacity, required a consultee during the discussion or who were under the age of 18 years.

Phase 2: staff feedback was collected through an online survey sent by email, containing 10 questions about the CDPR form and implementation (https://www.surveymonkey.co.uk/r/YZ2JVVZ). All survey responses were confidential and anonymous. Staff were given 1 month to complete the questionnaire, with non-responders receiving two email reminders.

Phase 3: three separate focus groups were held in meeting rooms at the clinical services (AA, MC and AMHT), which were familiar to the participating patients. Focus groups were facilitated by a researcher (SW) and a member of the clinical team. Written informed consent was obtained for all participants, and the focus groups were digitally recorded. The groups lasted no more than an hour, refreshments were provided and participants were reimbursed £20 for participation, plus travel expenses.

### Analysis

Phase 1: the UK-CRIS team pulled summary data and ran reports on the data held in EPR to establish the total number of patients attending each pilot site, the number of CDPR forms completed and the number of opt-ins. The age, gender and clinical diagnosis for these patients were obtained.

Phase 2: data analysis was conducted using Microsoft Office Excel 2013 calculating frequency and corresponding percentages to describe the responses to the survey questions. The Likert scale was dichotomised for the purposes of the analysis. A rating of either ‘very easy’ or ‘easy’ was regarded as a positive answer, ‘somewhat easy’ was regarded as a neutral answer and ‘difficult’ or ‘very difficult’ was viewed as a negative answer.

Phase 3: the focus group recordings were transcribed by SW; data were thematically analysed and managed using the Framework approach.^7^ Initial codes and categories were grouped and regrouped until themes began to emerge, and meetings with SW, CH and JP served as a means of triangulating the data by ensuring consistency and agreement of the final themes.

## Findings

### Phase 1

**Figure 1:**
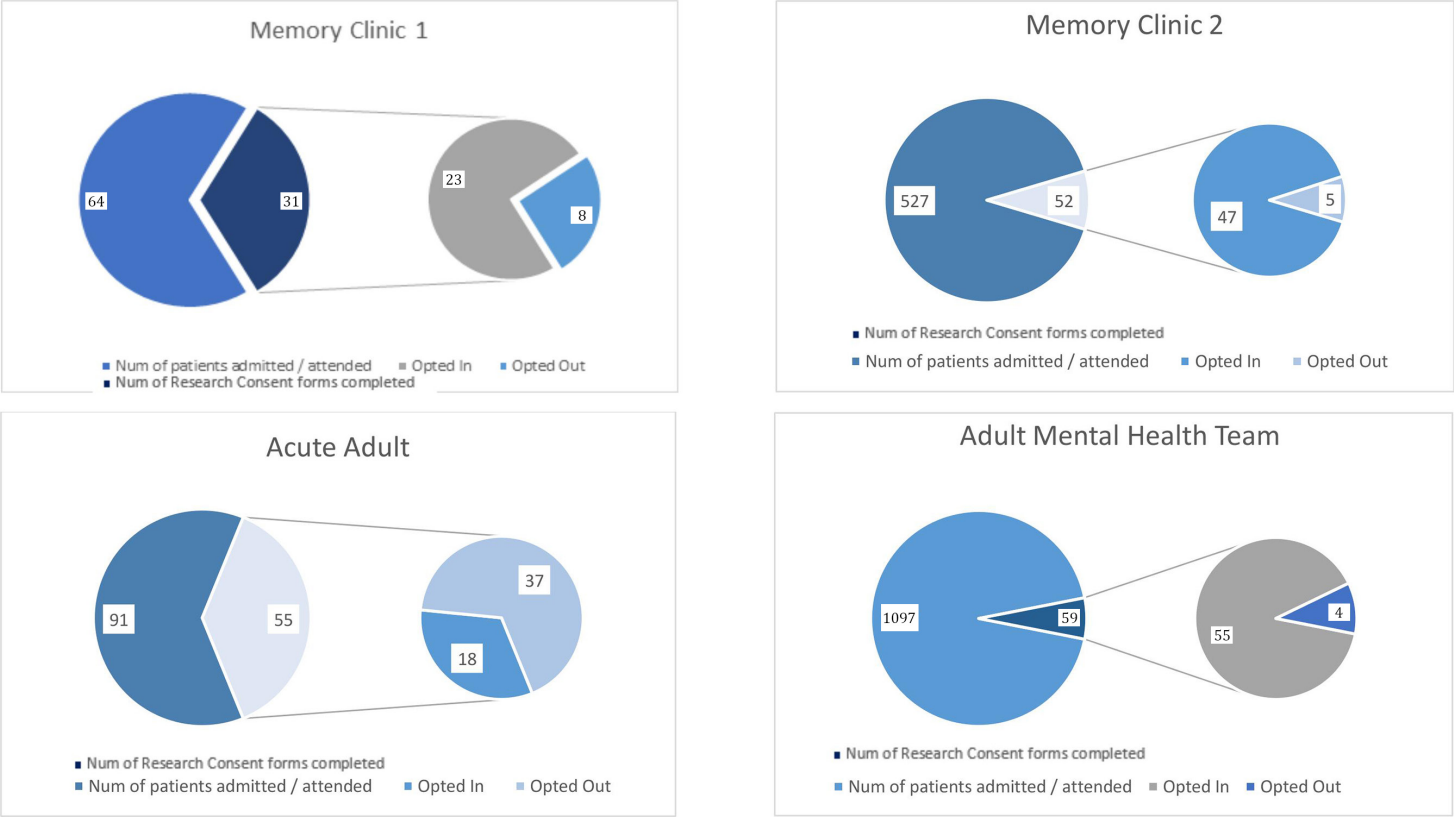
Number of CDPR forms completed and not completed for total number of patients attending each pilot site from July to December 2018. In each box, the large slice of the pie chart on the left shows the number of patients attending each pilot site over 6 months. The small slice represents the number of research consent forms completed by these patients. The pie chart of the right illustrates how many patients who completed research consent forms opted in (left side) and opted out (right side).

The number of patients attending each pilot site and the relative number of CDPR forms completed is summarised in figure 1. Across all four pilot sites, 1779 patients attended services during the pilot period. Of these, only 197 (11%) had a CDPR form completed by staff and 143 (8%) opted-in to being contacted about research. Table 1 shows the age, gender and diagnostic information for patients attending pilot sites and for patients who completed CDPR forms.

**Table 1:** Patient through-put, number of CDPR forms completed number of opt-ins at each pilot site between July and December 2018

Team name	Number of patients approached	CDPR forms completed (%)	Opt-ins (%)
Memory clinic 1	64	31 (48.44)	23 (35.94)
Memory clinic 2	527	52 (9.87)	47 (8.92)
Acute adult	91	55 (60.44)	18 (19.78)
Adult mental health team	1097	59 (5.38)	55 (5.01)
*Total*	1779	197 (11.07)	143 (8.04)

CDPR, consent to discuss their participation in research.

Form completion was not evenly spread across the staff who participated in the pilot. At the AMHT, one staff member (clinical research assistant) completed 38 out of 53 (72%) forms; of these, 97% patients opted-in. At another MC pilot site one staff member (nurse) completed 32 out of 68 (47%) forms; of these, 75% patients opted-in.

### Phase 2: summary of staff survey feedback

The questionnaire was sent to 47 members of staff who had taken part in phase 1, and the overall response rate was 27 (57%). Approximately, 17 (63%) were senior clinicians, 6 (22%) were junior clinicians, 3 (11%) were research staff and 1 (4%) was a member of the administration team. Table 2 shows the staff ratings of the CDPR form, the user experience of EPR and staff views on implementing the opt-in approach into clinical practice.

**Table 2:** Summary of staff feedback (total number of responses=27)

		Positive N (%)	Middle/indifferent N (%)	Negative N (%)
*CDPR form*	How familiar are you with what CDPR is?	14 (51.85)	9 (33.33)	4 (14.81)
	Do you think that the CDPR form is clear to use? (ie, which parts are compulsory to fill in)	13 (48.14)	6 (22.22)	8 (29.63)
	Do you agree with any of the following statements:		
	The form contains too much information.	1 (3.85)		
	The form is too long.	2 (7.69)		
	Iti s not clear what needs to filled out and what can be left blank.	9 (34.62)		
	The capacity assessment is confusing.	4 (15.38)		
	It is not clear whether to save or confirm the form.	9 (34.62)		
	None of the above,	11 (42.31)		
*Electronic patient records (EPR*)	Is the form easy to find on EPR?	19 (70.37)	5 (18.52)	3 (11.11)
	Would prompts in EPR/other reminders help you to remember to fill in the form?	20 (74.08)	5 (18.52)	2 (7.41)
*Implementing into routine practice*	How easy did you find implementing this form into your routine practice?	12 (44.44)	14 (51.85)	1 (3.70)
	Do you agree with any of the following statements?		
	Hard to identify when it is most appropriate to ask the patient.	4 (14.81)		
	Hard to get the whole team to use the form, not just one dedicated person.	13 (48.15)		
	Not a priority/lack of interest from staff.	10 (37.04)		
	Lack of interest or understanding from patients.	3 (11.11)		
	Lack of support and resources to trial using the form properly.	2 (7.41)		
	None of the above.	8 (29.63)		

CDPR, consent to discuss their participation in research.

### Phase 3: summary of main themes from patient focus groups

Overall, 18 patients participated in the focus groups. Their average age was 48.9 years, and the sample was predominantly male (72%) and white British (89%). Patient summary characteristics are presented in table 3.

**Table 3:** Summary characteristics of focus group participants

	Memory clinic Patient: n=5 Carer: n=4	Acute adult n=4	Adult mental health team n=5
Age in years (range)	Patient: 68.1 (64–79) Carer: 65.4 (57–68)	42.3 (34–47)	37.7 (18–41)
Gender			
Male	Patient: 4; carer: 0	4	5
Female	Patient: 1; carer: 4	0	0
Ethnicity			
Caucasian	9	4	3
Other	0	0	2
Clinical diagnoses			
F00-F09: organic, including symptomatic, mental disorders (includes dementia)	Patient: 5; carer: 0	0	0
F20-F29: schizophrenia, schizotypal and delusional disorders	0	4	2
F30-F39: mood (affective) disorders (includes depression and bipolar)	0	1	1
F60-F69: disorders of adult personality and behaviour	0	1	2
Participated in research			
Yes	0	0	3
No	9	0	2

The main themes to emerge from the data set about the CDPR pilot were ‘patient choice’, ‘trust in the system’, ‘respect for patient journey’, ‘perception of research’ and ‘recruitment efficiency’.

#### Patient choice

Some participants said they would immediately know how to respond to the opt-in question, while others said they would prefer time to consider it and might want more information about what taking part in research would involve and the types of studies on offer; a few participants indicated that the clinic environment might not be the best setting for gathering this information:

I wouldn’t say yes or no straight away, I would want to know more and then have a think – you might need some time and then come back. (AMHT)

I’d feel confident to say no at any time, but it might be nice to think about it at home and then answer. (Memory clinic patient (MCP)).

Would be happy to receive a text/letter, with a number to call if you wanted to ask questions/get more info. (AA)

Participants generally did not think the choice to opt-in should ever be made by a consultee if they did not have capacity:

I would never pick someone else to answer the question. No it’s me or that’s it. (AMHT)

#### Trust in the system

Participants trusted some methods of research contact over others and stated a preference for this to be recorded on their CDPR form. The majority preferred written contact via an email or postal letter:

We get so many people calling us trying to sell solar panels - it’s important for you to say who you are and what you want really quickly before I put the phone down - maybe a written contact is better. (MCP)

Participants did not like being contacted by phone due to an intense dislike for calls from unknown numbers:

Don’t like random unknown numbers. Being 'cold called' by a researcher is no [better] than being stopped by a chugger on the street! (AA)

The reasons for disliking unknown contact from researchers by phone depended on the clinical presentation of the patient. In the case of the MC patients, they stated it was because they were worried that they would forget if they had opted in or not. In the case of the AA and AMHT patients, it was reportedly due to paranoia:

Makes me sceptical of who is it so I’d just decline it. When I get private numbers it makes me nervous especially on bad day and feeling really paranoid. (AMHT)

Patient preferences about which staff member asked them the CDPR question was based on the level of trust they had with different staff members. The MC group had high levels of trust in the memory service and ambivalent to who they were asked by, whereas inpatient participants reported distrust in clinicians (due to being under section or medication disputes) so preferred being asked by a support worker. The AMHT group indicated that they would be more likely to opt-in if they were approached by a trusted healthcare professional.

I see my GP more often than coming to AMHT now – so a trusted GP is better because the setting is better, it’s more regular and consistent. (AMHT)

#### Respect for patient journey

The timing of being asked the CDPR question was important in terms of respecting when the patient’s clinical needs take priority over research and generally during clinical assessments was seen as inappropriate:

I don’t think the assessment is the best time - you’re finding out that you’ve just got a diagnosis of MCI and all this is going on, for me it was upsetting. (MCP)

AMHT and inpatients said that the instability of their admission and treatment periods meant the CDPR question would be best asked at discharge:

At the beginning when I was having serious problems that would have been prioritised above talking about research. (AMHT)

The ward is when you're not in a good place. You should be asked the question at your discharge session because that it when you are in the best place. (AA)

#### Perception of research

There was limited understanding about the links between research and improved treatment and clinical care, but there was general consensus that research and improving recruitment was important.

Research is really important, otherwise we’d never get anywhere new. If people are given opportunities to be involved in it I think it’s a really good thing. (MC-C)

However, AMHT and AA participants had more negative perceptions of research as invasive and highly medicalised:

Research is what the Nazi’s did on people - I don’t want to be a guinea pig! (AA)

Most participants did not think that research participation should be altruistic and that it should be incentivised, either financially or through a guarantee of improved clinical outcome, given the potential risks of taking part or being on placebo:

What’s the point in taking part in research if there are irreversible side effects or you’re on the sugar pill? (AA)

#### Recruitment efficiency

Participants highlighted the need for research contact to be efficient by maintaining up to date records of their personal information on EPR:

It’s about keeping up with the volunteer group – dynamics of it are always moving - people move away, die, don’t want to continue – so that you don’t waste your time contacting people who aren’t suitable anymore. (MCP)

After discharge to the community team, you might be moved on twice so your address will change or you stay with family. (AA)

For MC participants this also included ensuring that the contact details of the patient’s carer were accurately recorded on EPR (with the patient’s consent), so that any research contact could involve them.

Some participants thought that being contacted about research was an uncontentious issue, so asking for consent to contact caused unnecessary complication and confusion. Many argued that although clinical staff did have time to ask the CDPR question, research recruitment could be more efficient if the question was removed altogether and patients were contacted directly by researchers. However, one participant on the inpatient ward disagreed:

You should ask this question otherwise people will be bombarded. (AA)

## Discussion

In our study, the majority of patients and staff viewed research very positively. The discordance between the generally positive views of the opt-in approach and the very low completion rate limits the feasibility of CDPR as a strategy to improve access to research for real-world patients. This study provides evidence that despite both patient and staff beliefs that research is important, there are still barriers to translating this into practice.

The barriers identified by staff relating to discussing research with patients included perceived lack of time, lack of awareness and interest in research and a poor user experience of the EPR system. These issues were not raised at monitoring visits, and the CDPR team only ever received two emails with queries from staff, indicating that they were not a priority to resolve. Previous research supports these findings that NHS staff are generally demotivated about research,^8^ and there is a lack of time for research activity,^9^ but these only relate to acute hospital trust settings.

The diversity of staff who participated in the study may help explain the differences between form completion at each of the pilot sites, as research-active staff were more confident talking about research and had allocated time to do so. For example, an assistant psychologist at an MC who already discusses research with patients is likely to find implementing CDPR more feasible than a healthcare support worker on an inpatient ward, where high acuity, staff handovers and quantity of clinical admin per patient is prioritised over research activity. Although the aim of CDPR was that all staff would ask the opt-in question, in reality, only a handful did so. This implies that protecting time for all clinical staff to discuss research with patients may be beneficial.^2^

The importance of NHS consumers being involved in decisions about how participants are recruited to research is well established,^10^ but to our knowledge, this is the first study of its kind to incorporate both patient and staff perspectives, a range of clinical settings and both qualitative and quantitative methodologies in solving recruitment challenges in a mental health trust setting.

Overall, our conclusion is that while modifications may be made to the current opt-in approach in order to improve its success, the approach is inherently inefficient. While this study only analysed one approach to research recruitment, alternative approaches exist, each with their own ethical considerations in relation to patient data protection and issues of coercion.^11^ The ethical and practical benefits of an opt-in approach are debatable, as it could be argued that this approach leads to only a handful of patients being offered opportunities to participate in research due to a lack of clinician engagement. In addition, this approach can lead to low response rates, wasted resources and research of limited scientific validity due to a lack of representation from the general patient population. Other approaches, such as a transparent opt-out approach, whereby patients are offered the opportunity to hear about research opportunities unless they indicate otherwise, should be considered and would increase the likelihood of obtaining representation from the general patient population, while still allowing patients to choose whether they wish to participate in research.^12^ Careful consideration of these different options is required to ensure that patients’ best interests are protected and that mental health patients are being offered opportunities to take part in research that might improve their healthcare outcomes.

## Clinical implications

The potential implications for practice from this study are significant; if all patients are provided with better access to research, both research and clinical outcomes can be improved. Research participants would be more representative of the clinical population in general, and this would strengthen the evidence base of treatment for all patients. If research awareness opportunities could be more comprehensively disseminated, this would reduce the requirement for clinicians to refer patients to research studies and would allow all staff to discuss research with their patients.

By improving the methods of recruiting patients to research studies, NHS Trusts can align with the priorities of institutions such as the National Institute for Health Research, which aim to improve the health and wealth of the nation through research.^13^ In addition, they can move towards a clinical service where all patients are properly informed about the benefits of taking part in research and are provided with equitable access to studies. Increasing opportunities for research participation would also serve to develop and equip staff with the research skills needed to drive research participation forward as their research and clinical practice environments become increasingly intertwined. This pilot study demonstrates that the evaluation of strategies is critically important as we continue to innovate and evolve more effective systems of embedding research into real-world practice. It is hoped that this study can generate new ideas and strategies for other NHS Trusts across the UK and internationally.
