# Supplementary material for: Consent to discuss participation in research: a pilot study
Source: Evid Based Ment Health. 2019 Sep 26;23(2):77–82. doi: 10.1136/ebmental-2019-300116 (PMC7229904; doi:10.1136/ebmental-2019-300116)
Supplement: Supplementary data [file ebmental-2019-300116supp001.pdf]

Appendix 1. Consent to discuss research participation (CDPR) form on EPR (screenshot)

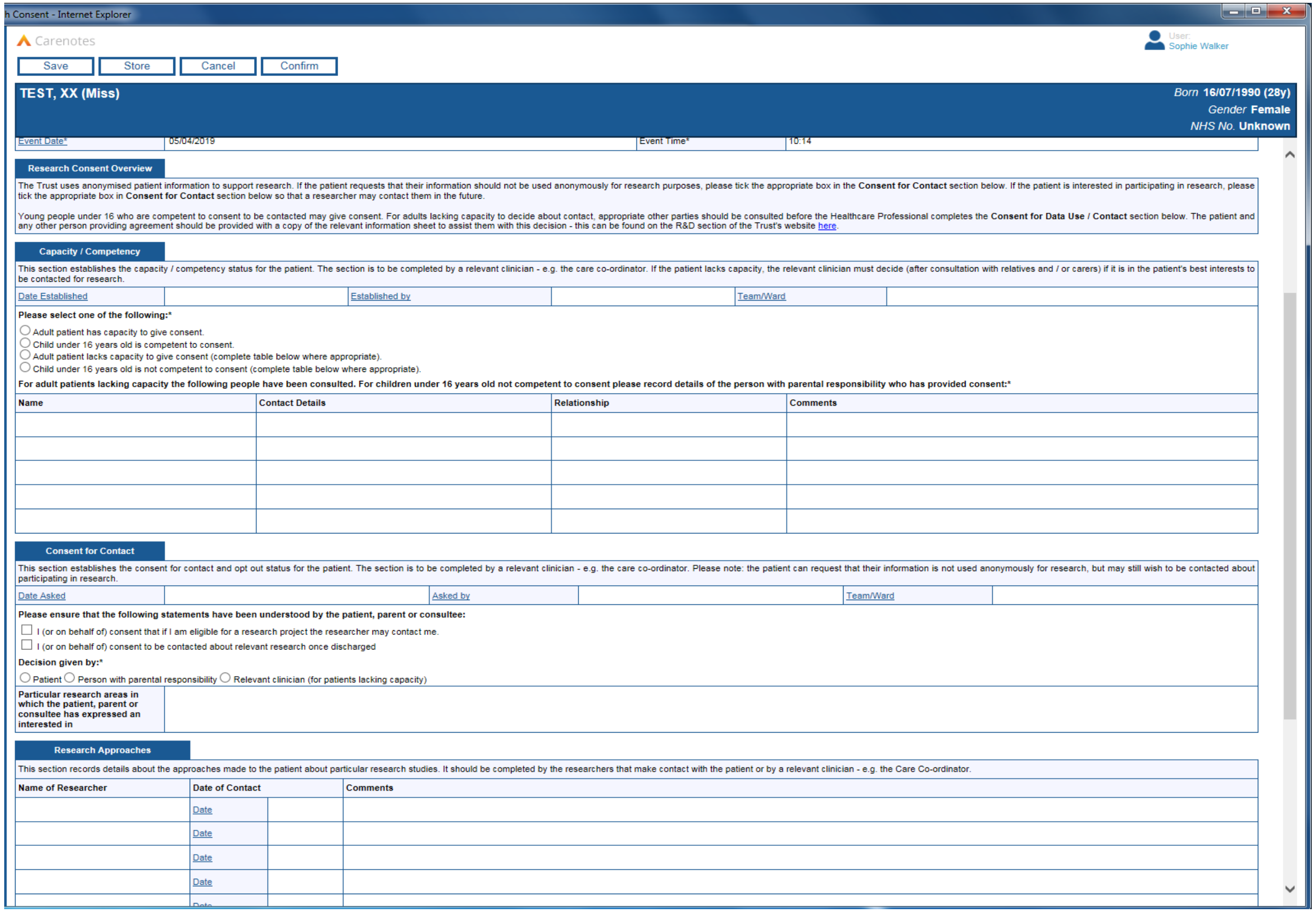
h Consent - Internet Explorer

Carenotes

User: Sophie Walker

Save | Store | Cancel | Confirm

TEST, XX (Miss)

Born **16/07/1990 (28y)**
Gender **Female**
NHS No. **Unknown**

Event Date*	05/04/2019	Event Time*	10:14

Research Consent Overview

The Trust uses anonymised patient information to support research. If the patient requests that their information should not be used anonymously for research purposes, please tick the appropriate box in the **Consent for Contact** section below. If the patient is interested in participating in research, please tick the appropriate box in **Consent for Contact** section below so that a researcher may contact them in the future.

Young people under 16 who are competent to consent to be contacted may give consent. For adults lacking capacity to decide about contact, appropriate other parties should be consulted before the Healthcare Professional completes the **Consent for Data Use / Contact** section below. The patient and any other person providing agreement should be provided with a copy of the relevant information sheet to assist them with this decision - this can be found on the R&D section of the Trust's website here.

Capacity / Competency

This section establishes the capacity / competency status for the patient. The section is to be completed by a relevant clinician - e.g. the care co-ordinator. If the patient lacks capacity, the relevant clinician must decide (after consultation with relatives and / or carers) if it is in the patient's best interests to be contacted for research.

Date Established		Established by		Team/Ward	

Please select one of the following:*

- ○ Adult patient has capacity to give consent.
- ○ Child under 16 years old is competent to consent.
- ○ Adult patient lacks capacity to give consent (complete table below where appropriate).
- ○ Child under 16 years old is not competent to consent (complete table below where appropriate).

For adult patients lacking capacity the following people have been consulted. For children under 16 years old not competent to consent please record details of the person with parental responsibility who has provided consent:*

Name	Contact Details	Relationship	Comments

Consent for Contact

This section establishes the consent for contact and opt out status for the patient. The section is to be completed by a relevant clinician - e.g. the care co-ordinator. Please note: the patient can request that their information is not used anonymously for research, but may still wish to be contacted about participating in research.

Date Asked		Asked by		Team/Ward	

Please ensure that the following statements have been understood by the patient, parent or consultee:

- ☐ I (or on behalf of) consent that if I am eligible for a research project the researcher may contact me.
- ☐ I (or on behalf of) consent to be contacted about relevant research once discharged

Decision given by:*

○ Patient ○ Person with parental responsibility ○ Relevant clinician (for patients lacking capacity)

**Particular research areas in which the patient, parent or consultee has expressed an interested in**	

Research Approaches

This section records details about the approaches made to the patient about particular research studies. It should be completed by the researchers that make contact with the patient or by a relevant clinician - e.g. the Care Co-ordinator.

Name of Researcher	Date of Contact		Comments
	Date		
	Date		
	Date		
	Date		
	[illegible]		
