# Supplementary material for: Consent to discuss participation in research: a pilot study
Source: Evid Based Ment Health. 2019 Sep 26;23(2):77–82. doi: 10.1136/ebmental-2019-300116 (PMC7229904; doi:10.1136/ebmental-2019-300116)
Supplement: Supplementary data [file ebmental-2019-300116supp002.pdf]

Date and version No: Version 2.1 04 July 2019

Study Title: Consent to discuss participation in research – pilot study

Internal Reference Number / Short title: CDPR

Date and Version No: Version 1.1 20 May 2018

**Chief Investigator:**	Professor Andrea Cipriani, Oxford Health NHS Foundation Trust/ University of Oxford
**Investigators:**	Sophie Walker, University of Oxford
	Jennifer Potts, Oxford Health NHS Foundation Trust
	Dr Catherine Henshall, Oxford Health NHS Foundation Trust/Oxford Brookes University
	Mike Denis, UK-CRIS
	Mark Hancock, Oxford Health NHS Foundation Trust
	Professor John Geddes, University of Oxford
	Professor Simon Lovestone, University of Oxford
**Sponsor:**	Oxford Health NHS Foundation Trust
**Funder:**	Biomedical Research Centre (Digital/Informatics theme)
**Chief Investigator Signature:**	

Confidentiality Statement

This document contains confidential information that must not be disclosed to anyone other than the Investigator Team, host organisation, unless authorised to do so.

© Copyright: Oxford Health 2018

Walker S, *et al. Evid Based Ment Health* 2020; 23:77–82. doi: 10.1136/ebmental-2019-300116

Date and version No: Version 2.1 04 July 2019

Table of Contents

1 SYNOPSIS 3
2 ABBREVIATIONS 3
3 BACKGROUND AND RATIONALE 4
4 OBJECTIVES 5
5 PILOT SITES 6
7 RECRUITMENT 6
8 CAPACITY AND CONSENT 7
9 STUDY PROCEDURES 7
10 DEFINITION OF END OF STUDY 7
11 ANALYSIS 7
12 DATA MANAGEMENT 8
13 QUALITY ASSURANCE PROCEDURES 8
14 ETHICAL AND REGULATORY CONSIDERATIONS 8
16 FUNDING 8

© Copyright: Oxford Health 2018

Walker S, *et al. Evid Based Ment Health* 2020; 23:77–82. doi: 10.1136/ebmental-2019-300116

Date and version No: Version 2.1 04 July 2019

1 SYNOPSIS

**Study Title**	Consent to discuss participation in research – pilot study	
**Internal ref. no. / short title**	CDPR	
**Study Design**	The CDPR Study will determine the feasibility of asking all clinical staff to complete a new form on Carenotes to enable all patients registered with Oxford Health NHS Foundation Trust the opportunity to take part in research.<br>The form records whether patients choose to opt-in to being contacted about research opportunities and can be filled in by any member of a clinical team, at any point during a patient's care in the Trust.	
**Pilot Sites and Study Participants**	Four pilot sites were selected to represent a range of clinical services and patients within the Trust: two memory clinics, an Adult Mental Health Team (outpatients), and an Acute Adult ward (inpatients). The number of Oxford Health staff at the four pilot sites can vary but all have access to Carenotes.<br><br>Study participants will be consecutive patients attending one of the four Oxford Health pilot sites during the six-month study period and will be recruited using a convenience sampling method.	
**Planned Study Period**	July 2018 - December 2018	
	**Objectives**	**Outcome Measures**
**Primary**	To determine the feasibility of using the CDPR form on Carenotes to record whether patients opt-in to being contacted about research opportunities.	It is expected that clinical staff will complete a CDPR form for the majority of patients attending clinical services during the six-month study.
**Secondary**	To collect feedback from patients and clinicians about the CDPR form.	At the end of the study, an online staff survey and patient focus groups will be used to identify ways to improve CDPR .

2 ABBREVIATIONS

BRC	Biomedical Research Centre
CDPR	Consent to discuss participation in research
CRIS	Clinical Record Interactive Search
NHS	National Health Service
OHFT	Oxford Health NHS Foundation Trust

© Copyright: Oxford Health 2018

Walker S, *et al. Evid Based Ment Health* 2020; 23:77–82. doi: 10.1136/ebmental-2019-300116

3 BACKGROUND AND RATIONALE

The third key principle of the NHS constitution is, 'The NHS aspires to the highest standards of excellence and professionalism… through its commitment to innovation and to the promotion, conduct and use of research to improve the current and future health and care of the population' (NHS, 2015). The NHS pledges to inform patients of research studies in which they may be eligible to participate in, and also pledges to anonymise the information collected during the course of treatment and use it to support research and improve care for others (NHS, 2015).

Patients who take part in research have improved clinical outcomes (Ozdemir et al, 2015), therefore improving access to research for all patients will improve the quality of research and clinical care for all. This is the mission of the National Institute of Health Research (https://www.northdevonhealth.nhs.uk/services/research/national-institute-health-research-research-campaign/). However, recruiting patients into research studies within the NHS can be challenging, with a number of barriers. It can be difficult for researchers to identify potential participants for specific studies, as they are not able to contact patients directly.

Researchers are largely dependant on clinician referrals in order to recruit participants for their studies, but this is often a time-consuming process for clinicians as they are required to understand protocols and inclusion/exclusion criteria in order to be able to identify suitable patients and make the referral to the research team. Clinicians who hold dual academic/clinical roles are more likely to make clinical referrals for research (Rahman et al, 2011; Lambert, Smith & Goldacre, 2015) , thus research participants often only represent patients from small numbers of clinicians, and are not representative of the general clinical population.

In order to improve access to research for all patients, all patients need to be properly informed and educated about the potential benefits of taking part in research, and they also need to be asked for their permission to be contacted about research opportunities. To improve on the current system, this process could be automated and needs to empower all clinical staff, not just clinicians, to talk to patients about research. In order to do this, instead of building something new to capture this consent, we propose building it into an existing system already used by the trust.

In Oxford Health, this system is the electronic patient health records system called Carenotes, which stores all patient data. By adding into Carenotes a new form with the CDPR question, the process will become automated because it is the same form on every patient's online Carenotes

© Copyright: Oxford Health 2018

Walker S, *et al. Evid Based Ment Health* 2020; 23:77–82. doi: 10.1136/ebmental-2019-300116

profile. It will also empower clinical staff, because all NHS staff have access to Carenotes, not just clinicians. South London and Maudsley NHS Foundation Trust have already implemented a similar approach using electronic patient health records, and now have over 17,000 patients opted in to hearing about research opportunities. 1,121 of these patients (6.6%) have gone on to participate in research.

The Oxford Health CDPR form on Carenotes will include a section indicating whether the patient had capacity to answer the CDPR question, and also a question about whether they would be happy to be contacted about research opportunities after they've been discharged from the trust.

This study is firstly to determine the feasibility of asking NHS staff to implement the use of the CDPR form on Carenotes into their routine clinical practice, and secondly to collect feedback from patients and clinicians about opting-in to research contact. The results of this pilot study will be analysed in order to identify where improvements can be made to CDPR. This evaluation will then form the basis for a decision about whether this opt-in approach is rolled out across the whole Trust or whether an alternative method of informing patients about research opportunities should be considered.

4 OBJECTIVES

1. To determine the feasibility of using the CDPR form on Carenotes to record whether patients opt-in to being contacted about research opportunities.

2. To collect feedback from patients and clinicians about the CDPR form.

It is expected that clinical staff will complete a CDPR form for the majority of patients attending clinical services during the six-month study. At the end of the study, staff feedback collected through an online survey will be used to understand the key factors relating to the implementation of the CDPR form into routine clinical practice. Patient focus groups will also be conducted with participants from each pilot site to understand the acceptability of asking patients to opt-in to research contact.

CONFIDENTIAL

© Copyright: Oxford Health 2018

Walker S, *et al. Evid Based Ment Health* 2020; 23:77–82. doi: 10.1136/ebmental-2019-300116

5 PILOT SITES

This feasability study will take place across four pilot sites within OHFT between July and December 2018. The four pilot sites were selected to represent a range of clinical services and patients within the trust; two memory clinics, an Adult Mental Health Team (outpatients), and an Acute Adult ward (inpatients). All pilot sites had the same clinical lead; a consultant clinician who was also experienced in conducting research with the Department of Psychiatry, University of Oxford. All pilot sites had a range of staff involved, but crucially all staff had access to the Carenotes system, which is used daily via desktop computers in services.

6 STUDY PARTICIPANTS

6.1 Inclusion Criteria

1. Any Oxford Health patient attending clinical services at each of the pilot sites for the duration of the six-month pilot study.

6.2 Exclusion Criteria

1. Any patient lacking capacity and requiring a consultee to answer the research question; the use of the consultee section will not be piloted in the present feasibility study.
2. Any Child and Adolescent Mental Health patient

7 RECRUITMENT

Patients will be recruited using opportunistic sampling at each pilot site by any member of the clinical team including clinicians, nurses, support workers and research assistants. Any member of the team may ask the CDPR question to a patient, and record their answer on the CDPR form on Carenotes. Therefore, the member of staff must have access to Carenotes.

Administrative staff may also complete the CDPR form on behalf on a clinical member of staff. Only members of the clinical team are intended to conduct the capacity assessment for CDPR.

Depending on the type of service (ie, inpatient versus outpatient), the clinical teams will be asked as part of the feasibility study to develop their own recuritment strategy; it is up to them to determine when and how it is most appropriate to ask their patients the CDPR questions. For example, some teams may prefer to ask patients at their assessment visit, others may prefer to ask it as the discharge visit when a patient might be better stabilised.

© Copyright: Oxford Health 2018

Walker S, *et al. Evid Based Ment Health* 2020; 23:77–82. doi: 10.1136/ebmental-2019-300116

8 CAPACITY AND CONSENT

This feasibility study requires clinical staff to assess patient capacity in order to ask the CDPR question. If a patient does not have capacity for this question at a specific time, ideally they should be asked on another occasion if the staff member thinks their capacity may return once treatment is stabilised.

9 STUDY PROCEDURES

The CDPR form on Carenotes will be used by clinical staff at each pilot site and used following local procedure agreed upon by the clinical team in question. Broadly though, the procedure involves a member of staff identifying a patient who has not been asked the CDPR question, conducting a brief conversation with them to ask if they are happy to be contacted about research and then to record their answer on the CDPR form on Carenotes.

10 DEFINITION OF END OF STUDY

The end of the study will be the 31st December 2018.

11 ANALYSIS

There will be three data collection phases; 1) completion of the CDPR form on Carenotes by pilot sites from July-December 2018, 2) completion of online staff survey in May 2019, 3) patient focus groups conducted in May-June 2019.

Phase 1. The Oxford-CRIS team for the NIHR Oxford Health Biomedical Research Centre, will run reports on the data held in Carenotes for the period July-December 2018, to establish the total number of patients attending each pilot site, the number of forms completed and the number of opt-in’s obtained during this period. The age, gender and clinical diagnostic information for these patients will also obtained.

Phase 2. Data analysis calculating the frequency and corresponding percentages to describe the responses to the survey questions. The Likert scale will be dichotomised for the purposes of the analysis. Answers to open questions were grouped according to theme to allow for analysis.

Phase 3. The focus group recordings will be transcribed and the data will then thematically analysed and managed using the Framework approach.

© Copyright: Oxford Health 2018

12 DATA MANAGEMENT

Data from the study will be stored on Carenotes, which is only accessible to OHFT staff. All information stored about patients on their Carenotes profile (including the CDPR form) is confidential but not anonymous. Carenotes is managed by the Carenotes Software Management Team at Oxford Health NHS Foundation Trust.

13 QUALITY ASSURANCE PROCEDURES

The study will be monitored throughout by the Study Coordinator based at Oxford Health, and working closely with the Chief Investigator. Each site will have a Site Initiation Visit, including study protocol training, before beginning study activities.

14 ETHICAL AND REGULATORY CONSIDERATIONS

The Investigator will ensure that this study is conducted in accordance with the principles of the Declaration of Helsinki. The Investigator will ensure that this study is conducted in accordance with relevant regulations and with Good Clinical Practice. The protocol, CDPR form on Carenotes, CDPR Patient Information Leaflet, CDPR PPI Participation Information Sheet and CDPR PPI Informed Consent Formdo not need to be submitted for ethical approval due to the status of the study being a service evaluation. Moreover, approval of materials will be reviewed by the Research and Development team in Oxford Health NHS Foundation Trust, including the Patient and Participation Liasion Team.

16 FUNDING

The study is funded through a grant from the Oxford Health Biomedical Research Centre (BRC).

© Copyright: Oxford Health 2018

Walker S, *et al. Evid Based Ment Health* 2020; 23:77–82. doi: 10.1136/ebmental-2019-300116
